# Risk factors for persistent airflow limitation: Analysis of 306 patients with asthma

**DOI:** 10.12669/pjms.306.5363

**Published:** 2014

**Authors:** Lingcheng Wang, Shuncui Gao, Wei Zhu, Jun Su

**Affiliations:** 1 Lingcheng Wang, MD, Department of Respiratory Medicine, Fourth People’s Hospital of Jinan City, Shandong Province, 250031, China.; 2 Shuncui Gao, MD, Department of Respiratory Medicine, Fourth People’s Hospital of Jinan City, Shandong Province, 250031, China.; 3 Wei Zhu, MD, Department of Respiratory Medicine, Fourth People’s Hospital of Jinan City, Shandong Province, 250031, China.; 4 Jun Su, MD, Department of Respiratory Medicine, Fourth People’s Hospital of Jinan City, Shandong Province, 250031, China.

**Keywords:** Risk factors, Persistent airflow limitation, Asthma

## Abstract

***Objectives*** *:* To determine the risk factors associated with persistent airflow limitation in patients with asthma.

***Method*** *s:* This study was designed and carried out in the department of respiratory medicine, fourth People’s Hospital of Jinan City, Shandong province, China between Jan 2012 and Dec 2012. Three hundred and six asthma patients participating in the study were divided into persistent airflow limitation group (PAFL) and no persistent airflow limitation group (NPAFL). The patients participated in pulmonary function tests and sputum induction examination. The clinical data including age, gender, onset age, disease course, smoking history, family history, regular corticosteroid inhalation, hospitalization history and presence of atopy were collected.

***Results*** *:* In 306 patients, 128 (40.5%) were included in PAFL group and 178(59.5%) in NPAFL group. Multivariate analysis demonstrated smoking (≥10 pack-years; OR, 7.1; 95% CI, 1.8 to 31.2), longer asthma duration (≥ 20years) (OR, 6.3; 95% CI, 1.7 to 28.5), absence of regular corticosteroid inhalation (OR, 3.5; 95% CI, 1.1 to 14.5) and neutrophil in induced sputum≥65% (OR, 1.8; 95% CI, 1.0 to 2.8) were independent risk factors for PAFL.

***Conclusions*** *:* Smoking, longer asthma duration and increased neutrophil in induced sputum are risk factors for PAFL, while regular corticosteroid inhalation is protective factor. Smoking cessation and regular corticosteroid inhalation may play an important role in preventing the occurrence of persistent airflow limitation group (PAFL).

## INTRODUCTION

Asthma is a common inflammatory disease of airways characterized by reversible airflow obstruction.^   1 ^ In most patients, the symptom of airflow obstruction can be completely reverse after treatment. However, the airway obstruction in some patients may be persistent after treatment, even after corticosteroid inhalation. The chronic, persistent airflow limitation (PAFL) is reported to occur in patients with asthma as a function of severity and/or duration of disease.^1^^,^^2^ In addition, the incidence of PAFL is very high. In the study of 1017 patients from Lee, 612 patients (60%) showed evidence of PAFL, and in the study of 136 patients from ten Brinke,^   3 ^ sixty-six patients (49%) had PAFL. Also, PAFL may result in a lower life quality, higher treatment cost and poor prognosis,^      4 ^ as a result, PAFL should be paid a high attention by physicians.

Some authors suggest that specific factors might accelerate this progressive decline in lung function and promote the occurrence of PAFL. In the study of 152 patients with severe asthma, ten Brinke advocated PAFL is associated with adult onset of the disease, airway hyper responsiveness and sputum eosinophilia.^   3 ^ Lee suggested that older age, male gender, black ethnicity, current or past smoking, aspirin sensitivity and longer asthma duration were risk factors for PAFL.^   1 ^ Modification of the factors causing PAFL may be critical in preventing morbidities in patients with severe asthma. While, some authors carried out studies to detect the risk factors correlated with PAFL, but the outcome is controversial or contradictory.^   3 ^ Some factors, such as smoking, were regarded as a risk factor to promote PAFL in Guerra’s^      5 ^ and Lee’s study,^      4 ^ while it was excluded in Vonk’s study.^      6 ^ The increased eosinophil in induced sputum was regarded as the only independent risk factor associated with PAFL in ten Brinke’s study,^   3 ^ while in Lee’s study, he didn’t find any correlation between the cell count of induced sputum and PAFL.^      4 ^ Consequently, up to date, the risk factors causing PAFL are still not complete clear.

Therefore, in the current study, we consecutively recruited 306 out-patients with asthma in pulmonary departments of our hospital. The objective of the current study was: (1) To analyze the clinical characteristics of patients with PAFL; (2) To determine the risk factors for PAFL, and help physicians make treatment strategies.

## METHODS

This study was designed and carried out in the department of respiratory medicine, fourth People’s Hospital of Jinan City, Shandong province, China, between Jan 2012 and Dec 2012. In the study, we consecutively recruited the out-patients who met the inclusion criteria from the department of respiratory medicine of our hospital.

The inclusion criteria was patients with bronchial asthma history for more than three months, and asthma was defined according to the American Thoracic Society criteria. The patients with other pulmonary disease such asbronchiectasia, pulmonary tuberculosis, pulmonary infection, malignant neoplasm and serious heart failure were excluded. The current study was approved by the ethical committee of our hospital and performed according to the principles of Helsinki Declaration. All the included patients gave informed consent.

***Procedures: ***At the time of patients’ entry into the study, the clinical characteristics including age, gender, onset age, asthma duration, smoking history, family history, regular corticosteroid inhalation, hospitalization history and presence of atopy were documented according to a detailed structured questionnaire.^   3 ^ The onset age was judged as accurately as possible to determine the duration of asthma. In case of uncertainty, the earliest respiratory symptoms were taken into account.^   3 ^

The included patients underwent three pulmonary function tests under stable conditions without asthma exacerbation at the time of enrollment (baseline) and at 3 and12 months after enrollment, among which at least one test was performed both before and after inhalation of 200 microgram of albuterol.^      4 ^ The parameters measured in pulmonary function tests is forced expiratory volume in 1 second (FEV1) /forced vital capacity (FVC) ratio. Patients were classified into two groups: PAFL group and NPAFL group (without PAFL), based on the results of pulmonary function tests. PAFL was defined as an FEV1/FVC ratio < 0.7 on all of three pulmonary function tests despite use of high-dose inhaled corticosteroids and long-acting b2-agonists.^1^^,^^7^ No PAFL was defined when an FEV1/FVC ratio > 0.7 was proved on at least one pulmonary function test.^      4 ^

Sputum induction examination were performed according to previous studies.^8^^,^^9^ The patients received 200µg of salbutamol before the induction. After the post bronchodilator spirometry they inhaled sterile hypertonic saline at increasing concentrations at room temperature via a pneumatic nebulizer with the output set at 0.35 ml/min. The duration of each inhalation was 5 minutes and the induction was stopped after expectoration of an adequate amount of sputum.^      10 ^ Induced sputum was analyzed immediately and the cell counting was performed.

***Statistical analysis: ***Statistical analysis was performed using SPSS17.0 (Chicago, IL, USA).The comparison of age, cell counting, asthma duration between two groups was performed using independent 2-sample t test. The comparison of categorical variables including gender, family history, hospitalization history, smoking history and atopy between two groups was carried out using Chi-Square test. Univariate analysis and multivariate logistic regression analysis were performed to find the correlation between variables and PAFL. Variables showing a significant correlation were included in the multivariate logistic regression analysis to determine the independent risk factors associated to PAFL. A p value less than 0.05 was considered to indicate statistical significance.

## RESULTS

In the current study, 306 patients with asthma were included. PAFL group consisted of 128 patients (40.5%) and NPAFL group consisted of 178(59.5%).The clinical characteristics of two groups are shown in Table-I. The patients in PAFL group had relatively older age (p=0.01, independent 2-sample t test) and longer asthma duration (p=0.02, independent 2-sample t test) than those in NPAFL group. In addition, PAFL group had more patients with smoking history (p = 0.00, Chi-Square test), and the less patients treated using regular corticosteroid inhalation (p = 0.03, Chi-Square test). There was no significant difference in gender (p=0.98, Chi-Square test), education (p=0.18, Chi-Square test), onset age (p=0.98, Chi-Square test) and family history (p=0.84, Chi-Square test) between two groups. The initial percent predicted FEV1 was significantly lower in the PAFL group than that in NPAFL group (P=0.000, independent 2-sample t test). In addition, the ratio of FEV1/FVC in initial and 3 or 12 months after enrollment was significantly lower in PAFL than NPAFL group (all the p value is 0.00, independent 2-sample t test).

The cell classification in induced sputum in two groups was listed in Table-II. Sputum induction examination was completed in 98 patients in PAFL group, and 110 patients in NPAFL group. In terms of the cell count, there was no significant difference in monocyte (p=0.11, independent 2-sample t test), lymphocyte (p=0.78, independent 2-sample t test), and eosinophil (p=0.07, independent 2-sample t test) between two groups, but the neutrophil in PAFL group is significantly higher than those in NPAFL group (p=0.01, independent 2-sample t test) (Table-III).

The risk factors associated with PAFL was listed in Table-II. Univariate analysis indicated that smoking (≥10 pack-years), asthma duration (≥20years), absence of regular corticosteroid inhalation, patient age (≥50 years old), hospitalization, atopic status and neutrophil in induced sputum ≥65% were significantly correlated to PAFL. While, in multivariate analysis, smoking (≥10 pack-years; OR, 7.1; 95% CI, 1.8 to 31.2), longer asthma duration (≥ 20 years) (OR, 6.3; 95% CI, 1.7 to 28.5), absence of regular corticosteroid inhalation (OR, 3.5; 95% CI, 1.1 to 14.5) and neutrophil in induced sputum≥65% (OR, 1.8; 95% CI, 1.0 to 2.8) were independent factors for PAFL (Table-III).

## DISCUSSION

In the present study, the FEV1 value was 49.61% predicted in PAFL group, but the value was 82.79% in NPAFL group. This indicates that most cases with PAFL belongs to severe asthma.^   1 ^ In addition, 128 patients were included in PAFL group and 178 in NPAFL group. The incidence of PAFL is 40.5%, which is relatively lower, compared with the study from ten Brinke^   3 ^ and Lee.^   1 ^ In the two above mentioned studies, the recruited patients were all severe asthma cases, which had higher incidence of PAFL. While in the current study, the patients were recruited from out-patients, including cases with mild, moderate or severe asthma. This may explain the lower incidence in the current study.

Up to date, the mechanism of PAFL is still not completely clear. Many factors may be correlated closely to its occurrence, while some viewpoints are contradictory and controversial. Airway remodeling is regarded as an important mechanism of PAFL. Airway remodeling results in thickening of the airway wall and leads to a component of fixed airway narrowing and airflow obstruction.^      11 ^ The influence of smoking on PAFL has been emphasized by many authors.^4^^,^^5^ According to the viewpoints of Churg, cigarette smoke may drive small airway remodeling by induction of growth factors in the airway wall,^      12 ^ which explains why smoking can promote PAFL. In the current study, we also found smoking is a risk factors for PAFL. Subsequently, smoking cessation should be the primary step to prevent PAFL.

**Table-I:** Clinical characteristics of two groups*.

**Characteristics**	**PAFL**	**NPAFL**	**P value**
Patients	128	178	
Age (year)	49.53±12.48	42.69±13.37	P=0.01
Gender(male/female)	57/71	79/99	P=0.98
Smoking history (%)	88 (68.8%)	46 (25.8%)	P=0.00
Asthma duration (year)	18.63±13.54	12.88±12.19	P=0.02
Education (College graduate, %)	46 (35.9%)	85 (47.8%)	P=0.18
Hospitalization history (%)	48 (37.5%)	39 (21.9%)	P=0.03
Family history (%)	37 (28.9%)	49 (27.5%)	P=0.84
Presence of atopy (%)	39 (30.5%)	87 (48.9%)	P=0.03
Onset age ( ≥25years old, %)	83 (71.5% )	116(65.2%)	P=0.98
Regular corticosteroid inhalation (%)	35 (27.3%)	80 (44.9%)	P=0.03
FEV1, initial, predicted %	49.61±17.89	82.79±15.03	P=0.00
FEV1/FVC, initial, predicted %	48.79±12.15	77.59±11.04	P=0.00
FEV1/FVC, 3 months after enrollment, predicted %	51.62±13.45	80.32±12.28	P=0.00
FEV1/FVC,12 months after enrollment, predicted %	52.17±11.79	80.78±13.17	P=0.00

* Data are presented as No. (%) or mean±SD.

**Table-II:** The cell classification in induced sputum in two groups (%, x±s).

**Cell in induced sputum**	**PAFL**	**NPAFL**	**P value**
Monocyte	25.43±15.92	28.28±16.64	P=0.11
Lymphocyte	2.27±1.98	2.42±1.28	P=0.78
Neutrophil	66.56±17.64	61.51±16.92	P=0.01
Eosinophil	5.73±8.59	7.78±11.86	P=0.07

**Table-III:** Risk factors associated with persistent airflow limitation.

	**Univariate analysis**		**Multivariate analysis**	
	**p value**	**OR (95% CI )**	**p value**	**OR (95% CI)**
Smoking (≥10 pack-years)	0.000	4.1(2.9-8.1)	0.012	7.1(1.8-31.2)
Asthma duration (≥ 20 years)	0.042	2.1(1.3-4.0)	0.023	6.3(1.7-28.5)
Absence of regular corticosteroid inhalation	0.021	1.8(1.2-4.5)	0.032	3.5(1.1-14.5)
Patient age (≥50 years old)	0.003	3.7(2.2-6.1)	0.876	2.3(0.8-12.9)
Hospitalization	0.004	3.8(1.9-5.9)	0.981	1.5(0.5-6.2)
Presence of atopy	0.004	0.8(0.3-1.7)	0.072	1.1(0.7-3.1)
Neutrophil≥65%	0.001	3.6(1.3-4.4)	0.027	1.8(1.0-2.8)

Moreover, we found the longer asthma duration is the risk factor associated with PAFL. While, Vonk suggested the development of irreversible airflow obstruction is caused by a longer delay in treatment rather than by a longer duration of the disease.^      6 ^ In our opinion, the close correlation between inflammation and airway remodeling has been confirmed in some literatures.^13^^,^^14^ The longer asthma duration may result in a long-term inflammation stimulation on airways, and aggravate the airway remodeling adversely. In addition, we found in NPAFL group, there was 44.9% of patients treated using regular corticosteroid inhalation, but the rate was as low as 27.3% in PAFL group. The multivariate regression analysis indicated that regular corticosteroid inhalation was a protective factor for PAFL. It is possible that anti-inflammatory effect of corticosteroid may prevent the airway remodeling, then decrease the occurrence of PAFL. The protective effect of regular corticosteroid inhalation may also be an explanation of the risk effect of longer asthma duration on PAFL.

In addition, in the cell count in induced sputum, there was no significant difference in monocyte, lymphocyte and eosinophil between the two groups, but neutrophil in PAFL group is significantly higher than those in NPAFL group. Also, the logistic regression analysis demonstrate that the increasing cell count in neutrophil is one of the risk factors to cause PAFL, our result is similar to Shaw’s study,^      15 ^ but inconsistent with ten Brinke’s or Lee’s study.^3^^,^^4^ In the study of 136 nonsmoking patients with severe asthma, ten Brinke found the increasing eosinophil is the only independent risk factor associated with PAFL. While, Lee didn’t find any correlation between cells count in induced sputum and PAFL. The difference in included cases may explain the difference among studies, while the detailed mechanism may need to be studied in the future.

In conclusion, we found the identifiable clinical and demographic characteristics including smoking, longer asthma duration and increased neutrophil in induced sputum are risk factors for PAFL, while regular corticosteroid inhalation is protective factor. Subsequently, smoking cessation and regular corticosteroid inhalation may play an important role in preventing PAFL.
